# The functional significance of fasciculation and repulsion in a computational model of axon growth

**DOI:** 10.1186/1471-2202-16-S1-P17

**Published:** 2015-12-18

**Authors:** Robert Merrison-Hort, Oliver Davis, Roman Borisyuk

**Affiliations:** 1 School of Computing and Mathematics, Plymouth University, Plymouth, Devon, PL4 8AA, UK; 2 Brighton and Sussex Medical School, Brighton, East Sussex, BN1 9PX, UK

## 

The neurons in the developing spinal cord of a Xenopus tadpole form a simple network that is able to produce behaviors such as swimming and struggling from an early stage. At present, however, the developmental processes that allow such networks to form are not well understood. We use computational models to shed light on which features of development are important for the formation of networks that can produce patterns of neuronal activity corresponding to a particular behavior. Previously, we have shown that a simple model in which axon growth is guided by sensitivities to gradients of chemical cues can produce axonal trajectories that closely match the available anatomical data [1]. Furthermore, when the synaptic connectivity derived from these generated axons is mapped onto a large scale physiological model, the resulting network responds to simulated "skin touch" input by generating a stable pattern of anti-phase rhythmic activity which resembles that seen in real tadpoles during swimming [2], as well as reproducing other experimental observations such as transient synchrony [3].

**Figure 1:**
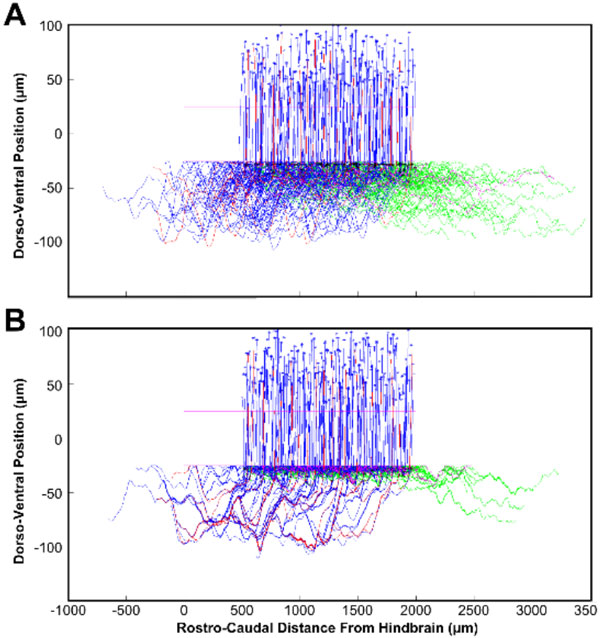
**Example trajectories of commissural interneuron axons from the original model (A) and with fasciculation (B)**. There are a small number of "pioneer" axons (red), which grow earlier than the main group (blue). Axons start growing from the somas (top, stars) and grow ventrally to cross the mid-line (dorso-vental position 0µm), before turning to grow in the ascending direction (rostrally). Every axon also has a secondary branch which grows in the descending direction (pioneers: magenta, others: green).

In this poster we demonstrate the effects of adding axon fasciculation and repulsion mechanisms to our growth model. Axon fasciculation is a process whereby a developing axon can detect the presence of another nearby axon and begin to grow along the existing axon; repulsion is the opposite process, where axons actively avoid each other. Both fasciculation and repulsion have been observed in the growth of commissural axons in the spinal cord of very early stage tadpoles [4], so we sought to use our computational model to investigate the possible function of these processes. The existing model was adjusted such that the growth angle of each axon contained an additional term based on the influence of nearby axons. This influence could be attractive or repulsive, and could vary based on the current position of the axon tip, since it appears that commissural axons change from repulsion to fasciculation after crossing the ventral mid-line. Adding these processes to the model caused dramatic changes to the pattern of axonal trajectories that were immediately visible (Figure 1), as well as quantitative changes to various anatomical characteristics, such as the total number of synapses formed. This poster shows the effects that fasciculation and repulsion have on both the generated anatomy and the dynamical behavior of the spiking neuron model, and discusses the possible biological and medical significance of these results (e.g. for axon regeneration).
